# Evaluating the Use of Contrast Sensitivity Tests By Orthoptists in the UK

**DOI:** 10.22599/bioj.317

**Published:** 2024-01-19

**Authors:** Lowri Jones, Anna O’Connor, Ashli Warburton

**Affiliations:** 1 Alder Hey Children’s NHS Foundation Trust, GB; 2 University of Liverpool, GB

**Keywords:** Contrast Sensitivity, Orthoptists, Contrast Sensitivity Testing

## Abstract

**Introduction:**

The importance of the use of contrast sensitivity (CS) tests in orthoptic practice is well established. However, despite the clinical relevance the implementation within clinical care is known to be variable. There are no known studies that investigate the use of CS tests in Orthoptic clinics in the UK, therefore the aim of this study is to gather information from Orthoptists in the UK on their opinion of CS and use of CS testing in clinical practice, now and in the future.

**Methods:**

An online survey was distributed via JISC to the British and Irish Orthoptic Journal newsletter three times over a period of four weeks in June 2021 inviting practising orthoptists in the United Kingdom to complete. The questionnaire comprised of a series of questions regarding current use with free text responses for additional information.

**Results:**

There were 84 responses to the survey. The preferred test for adult and children testing is Pelli Robson with 50% reporting use of this test. 56% felt there is a need for a new CS test for young children, 12% said no and 32% were unsure. The highest percentage (57.1%) of participants were confident to some degree that their preferred test gave them useful clinical information.

**Conclusion:**

The result of the survey demonstrates the variability of CS testing currently in orthoptic practice in the UK. It also highlights the lack of currently available tests for children for CS testing, which may be addressed by the addition of the new Double Happy CS test.

## Introduction

Visual Acuity (VA) is considered the gold standard for the evaluation of visual function in a clinical setting (Vingopoulos et al. 2021), which is usually tested in high contrast, using black optotypes on a white background. However, contrast sensitivity (CS) can be impaired in the presence of normal VA (Haegerstrom-Portnoy et al. 2000). Brabyn et al. (2000) described elderly participants who had good VA, but their VA reduced to 6/60 or less when low contrast conditions were introduced. The discrepancy between VA and CS can occur in a range of conditions such as optic pathway glioma, myopia, glaucoma, age-related macular degeneration, early diabetic retinopathy, and ocular hypertension (Regan and Neima 1984; Roy et al. 1997; Liou and Chiu 2001; Chang et al. 2007; Vingopoulos et al. 2021). Subtle reduction in CS may be apparent with these conditions before patients show reduced VA on testing (Jindra and Zemon 1989), highlighting the need for both aspects of visual function to be assessed, impacting on the diagnosis and management.

Evidence suggest that CS may be a better indicator of functional ability than VA testing. For example, in low-vision patients CS was determined to be a better indicator of reading performance than distance VA, where a slight reduction in contrast level of reading text resulted in a significant reduction in reading performance (Brown 1981). Similar results have been found by other studies which has found an association between reduced CS and reduced reading performance (Brussee et al. 2017; Brussee et al. 2018).

Other studies have highlighted the importance of the assessment of CS as defective CS can reduce task performance such as reading and studying, recognising faces and ability to use stairs and driving (Haymes, Johnston and Heyes 2002; West et al. 2002; Owsley 2003; Crossland et al. 2005). This link between impaired CS and functional ability has also been demonstrated using quality-of-life measures which have been found to be strongly correlated with CS (Hazel et al. 2000). Vingopoulos et al. (2021) determined that in some cases of age-related macular degeneration, CS is affected before VA and CS correlated better than VA with vision related quality of life. In addition, Roh et al. (2018) found that CS had a stronger association with a quality-of-life questionnaire compared to VA testing. Contrast sensitivity results can provide information to determine the impact of a condition, such as Cerebral Visual Impairment (CVI), nystagmus and retinopathy of prematurity (CRYO-ROP Group, 2001; Hertle and Reese, 2007; Good et al. 2012).

There is a range of available CS tests which vary in their presentation. For example, Cambridge Low Contrast Test (Ostadimoghaddam et al. 2014) and CamBlobs (Robson et al. 2019) are grating tests with fixed spatial frequency (SF) whereas Pelli Robson (PR) (Pelli et al. 1988), Mars CS test (Haymes et al. 2006) and Hiding Heidi (HH) (Leat and Wegmann 2004) is an optotype test with fixed SF and reducing contrast levels. Lea Symbols Translucent Low Contrast Chart (found on https://www.leatest.com/) has a variable SF as the contrast remains constant whilst the optotypes reduce in size. Vistech CS test is an example of a test were both the SF and contrast varies (Fitzgerald, 1989).

Pelli Robson appears to be the most used and accepted test for clinical testing of CS function (Gupta et al. 2017; Vingopoulos et al. 2021) with a high repeatability of 92–94% (Firdous and Sarfraz 2016) and reported to be a good measure of low to medium SF (Leat and Woo 1997). However, this test cannot be performed by patients who are unable to name letters, such as young children or adults with learning difficulties (Milling et al. 2014).

Although the importance of testing CS is well established, it often goes untested (Latham 1998; Vingopoulos et al. 2021). The lack of testing has been attributed to factors such as practical constraints, lack of available equipment and limitation of current tests (Latham 1998; Thayaparan et al. 2007; Vingopoulos et al. 2021). Some limitations of current tests include PR needing even illumination across the entire chart and having limited versions of the chart which impacts on measures of repeatability (Thayaparan, et al. 2007; Rijal et al. 2021). A limitation of LEA low-contrast symbols (LLCS) and Hiding Heidi (HH) is, that they overestimate CS, demonstrating a floor effect and not having a contrast level sufficiently low enough to test CS to thresholds in children with normal VA (Leat and Wegmann, 2004).

A questionnaire on the use of CS tests by UK optometrists (Latham 1998) found that only 16% (n = 75) of optometrists reported to assess CS. Since this study was published, the evidence supporting the use of CS testing has expanded considerably (Owsley 2003; Milling et al. 2014) and the role of orthoptists in many areas of ophthalmology has also grown. Orthoptists see a wide range of patients who experience deficits in CS, due to their involvement in clinics such as neurology, glaucoma and special educational needs (SEN). There are no known studies that investigate the use of CS tests in orthoptic clinics in the UK. Therefore, the aim of the study is to gather information from orthoptists in the UK on their opinion of CS and use of CS testing in clinical practice, now and in the future.

## Methods

The research followed the tenets of the Declaration of Helsinki and ethical approval was obtained from the University of Liverpool (reference 8018). An online survey was distributed via JISC to the British and Irish Orthoptic Journal newsletter three times over a period of four weeks in June 2021 inviting practising orthoptists in the United Kingdom to complete it. In addition, it was distributed via personal contacts and social media (Twitter). All participants consented to take part in the study, responses were anonymous.

The survey focused on the use of current CS tests for adults and paediatrics, preferences of tests and clinical confidence. It also asked for opinions of whether they perceive there is a need for a new paediatric test and opinion on aspects of the design. The questions can be found in Table 1. Data was extrapolated from JISC and stored and analysed using Excel and descriptive statistics were used to summarise data. The free text responses were grouped into common themes to be analysed.

**Table 1:** Survey questions.


QUESTION	RESPONSE OPTIONS

What CS test(s) do you have in your department?	Multiple choice: PR- paper format PR- computer format CSV-1000 Cambridge Gratings Low contrast acuity-test None Other (free text) Additional comments (free text)

What other CS test have you used previously?	Multiple Choice: PR- paper format PR- computer format CSV-1000 Cambridge Gratings Low contrast acuity-test None Other (free text) Additional comments (free text)

What is your preferred CS test for adult and paediatric testing?	Free text response Option for additional comments

How confident are you that your preferred test gives you useful clinical information?	Free text response Option for additional comments

What conditions would you assess CS in?	Free text response Option for additional comments

Do you test CS in Children?	Yes If yes, which test do you use? (Free Text) No If no, why? (Free text) Option for additional comments

Do you think there’s a need for a new CS test for young children?	Yes No Don’t know If yes or no, please explain why (Free Text) Option for additional comments

There are a number of ways to assess CS. Often in clinical practice, CS is commonly assessed with a chart of a fixed SF and a reducing contrast (to determine the contrast threshold), or by using a reducing SF chart at a fixed low contrast. If a new test was developed for children, which of the following would be your preferred format?	Multiple Choice: Pictures with constant SF with reducing contrast (Pelli Robson format) Pictures with constant contrast with reducing SF (same as logMAR VA test at a low contrast) Gratings with fixed SF but reducing contrast Gratings with fixed low contrast but reducing SF Additional comments

If you chose a constant contrast as your preference, what contrast level(s) do you think would be best?	Multiple choice: >10% 10% 5% 2.5%

Are you involved in any extended role clinics? If so, please select all that apply.	Multiple choice: SEN Retina SPLD LVA Stroke Neuro Additional comments

Do you have any other comments regarding the assessment of CS in children?	Free text only


Abbreviation: SPLD (Specific Learning Disability), LVA (Low Vision) Neuro (Neurology).

The participants were advised that the survey should take no more than 10 minutes.

## Results

There were 84 respondents from the survey, all of which could be included in the analysis. Participants had to complete every question before proceeding, except the last two questions which related to specific job roles. Not all answers were relevant to the questions asked therefore, some responses to questions had to be eliminated. For example, a response of ‘?’ to a question. All responses which were eliminated is listed in the results as ‘not relevant to the question asked’.

### Current use and opinion of CS tests

**Figure 1:**
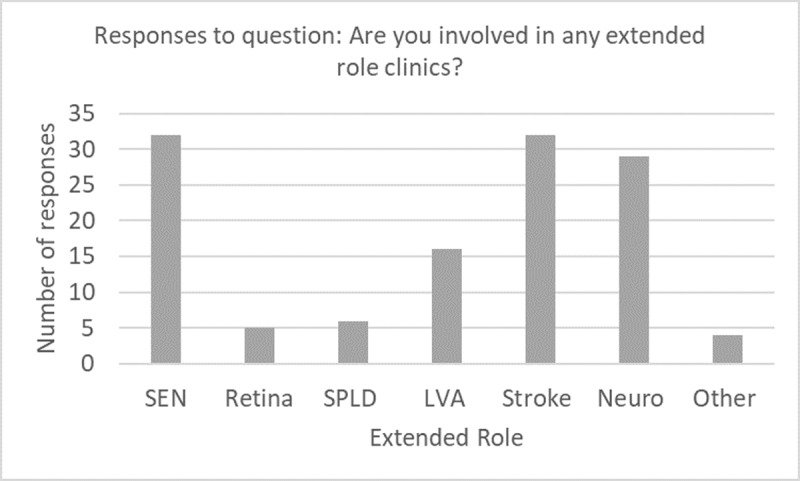
Bar chart demonstrating the responses to the question: Are you involved in any extended role clinics? Select all that apply.

In response to asking what extended roles orthoptists are currently involved in, the responses showed 32 were involved in SEN, 32 stroke and 29 neurology, with a range of other specialities as well, as shown in Figure 1. ‘Other’ includes falls, optometry, paediatrics, and uveitis extended roles.

Pelli Robson was the test used most currently or previously (Table 2). The tests listed under ‘other’ were HH, The Mars Letter CS Test, Low Contrast Flip Book using Lea/Sloan letters, Cardiff Cards, Spot Checks CS Chart (also known as CamBlobs2) (Rijal et al. 2021), Vistech Charts (Ginsburg and Evans, 1984), Berkeley Discs CS Test (Bailey et al. 2011) and Evans Letter Contrast Test (Amirkhanian et al. 2018).

**Table 2:** Number of responses to each test named from 3 questions:


TEST NAME	CS TESTS AVAILABLE IN DEPARTMENT	CS TESTS PREVIOUSLY USED	PREFERRED CS TEST FOR ADULT AND PAEDIATRIC TESTING

PR-paper format	43	44	50

PR- computer format	16	9	7

CVS-1000	2	3	5

Cambridge gratings	1	9	6

Low contrast acuity test	10	1	6

Other	18	11	8

None	15	24	8


What CS test(s) do you have in your department? What other CS tests have you used previously? What is your preferred CS test for adult and paediatric testing?

**Figure 2:**
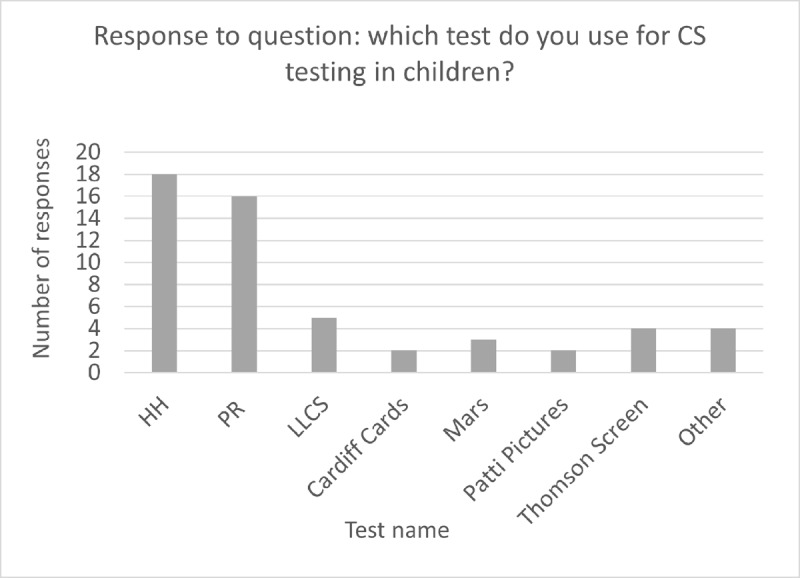
Bar chart demonstrating the responses: Do you test CS in children? If yes, which one?

47.6% of participants said they test CS in children. The tests used for testing CS in children is found in Figure 2. ‘Other’ included: Lea Numbers, Cambridge, Spot Checks and Berkely Discs. Several participants in the free text responses commented that they only use PR if the child is confident with letters and upper-case letters.

For the participants who said they did not test CS in children, the following reasons were provided as to why:

- n = 9 reported no CS tests available in clinic
- n = 7 reported lack of suitable CS tests available for children
- n = 9 reported CS testing wouldn’t add any useful information to their assessment
- n = 3 reported due to time constraints
- n = 2 reported due to seeing adults only
- n = 2 reported due to cooperation of children
- n = 4 reported they don’t test it as it isn’t done routinely in their department/practice
- n = 8 other reasons
- n = 1 response not related to the question asked

Other reasons were not part of assessment protocol, HH used as a vision assessment rather than CS, not known when needed, never thought to test it, out of practice, unable to keep uniform level of brightness in room, lack of access to room that has a PR chart and not sure of accuracy of CS tests.

The highest percentage (57.1%) of participants were confident to some degree that their preferred test gave them useful clinical information (Table 3). A few participants (n = 5) also commented that they did not feel CS adds any useful information to an ophthalmic investigation.

**Table 3:** Responses (%) (n = 84) to question: How confident are you that your preferred test gives you useful clinical information?


	VERY CONFIDENT (1)	CONFIDENT (2)	FAIRLY CONFIDENT (3)	NOT CONFIDENT (4)	UNSURE (5)	NOT APPLICABLE	RESPONSE NOT RELATED TO QUESTION ASKED

How confident are you that your preferred test gives you useful clinical information?	10.7	27.4	19	7.1	15.5	6.7	13.6


Two participants provided a free text response commenting on their confidence in the results from CS testing. Concerns were raised regarding the cleanliness and standardisation of the chart in one case, whereas another participant reported that they were confident due to the chart being wall mounted with static illumination. Further participants (n = 6) commented they lacked confidence due to lack of current literature or knowledge regarding normative values.

Patients with the following conditions were most likely to have their CS assessed: thyroid conditions, neurological conditions and ophthalmology conditions (Table 4).

**Table 4:** Summary of the responses to question: What conditions would you assess CS in?.


CONDITION	NUMBER OF RESPONSES

Thyroid conditions	24

Neurological Conditions	48

SEN	8

CVI	8

Ophthalmic condition: Anterior segment of the eye e.g., cataract and corneal opacity	13

Ophthalmic condition: Posterior segment of the eye e.g., Macula condition, retinal issue.	30

Amblyopia	8

Low VA	14

Normal VA with visual symptoms	4

Other	12


The conditions listed as ‘Other’ was functional visual loss, diplopia, falls patients, paediatric oncology patients, nystagmus, visual stress, visual perceptual difficulties, neurofibromatosis, and craniofacial disorders.

### Opinion on future development of CS tests

Fifty-six percent of participants felt there was a need for a new CS test for young children, 32% were unsure and 12% said no.

From the participants who indicated there was a need for a new CS for children, the most frequent responses were:

- Current paediatric tests unreliable/limited/not suitable, particularly for children with SEN.
- Need of a test for children who are too old for HH but not confident with letters yet.
- PR not suitable until children are older and able to name letters.

From participants who answered that they felt there was no need for a new CS test for children, the most common response was that it is not clinically useful to test CS and the assessment would not add any important information to the investigation.

For the response to question: ‘If a new test was developed for children which of the following would be your preferred format?’ 63% of participants preferred pictures with constant SF with reducing contrast, 21% participants preferred pictures with constant contrast with reducing SF, 13% of participants preferred gratings with fixed SF but reducing contrast, 3% of participants preferred gratings with fixed low contrast but reducing SF.

When asked to select a constant contrast for future tests, the highest percentage of participants preferred a constant contrast of 10% (Table 5).

**Table 5:** Table demonstrated the response to question: If you chose a constant contrast as your preference, what contrast level(s) do you think would be best? Please select as many as you think appropriate.


CONTRAST LEVEL	RESPONSES (%)

>10%	18.4

10%	35.9

5%	25.2

2.5%	20.4


## Discussion

The results of the survey sent to UK orthoptists indicated that the PR is the CS test that most respondents in the UK have, with 51% using paper format and 19% using the electronic format. Past studies have shown PR to have high reliability, repeatability (Elliott et al. 1990; Rubin 1988; Zimmerman et al. 2011), ease of use and testability on patients (Richman et al. 2013), supporting its use in clinic.

While survey responses showed acceptance of the PR for adults, 56% felt there was a need for a new CS test for young children. Tests available for use in young children and individuals with SEN are limited, the most reported being; LLCS and the HH (Chen and Mohamed 2003; Leat and Wegmann, 2004). Both tests have been determined to have poor agreeability with PR and HH measures CS 0.23 logCS higher in comparison to PR (Chen and Mohamed 2003; Leat and Wegmann 2004). In 30 adults, Chen and Mohamed (2003) found the results of HH and PR to have a positive correlation with each other (r = 0.65). However, there was a statistical difference in the results of the two tests (P < 0.01).

To address the issues of poor correlation with the PR, Mayer et al. (2020) designed a new CS test for young children or those with limited cognitive abilities, called the Double Happy (DH) CS test. Double Happy is based on forced choice preferential looking procedures. This test has been successfully used in a paediatric population with CVI and ocular disorders (Mayer et al. 2020) where 43 participants (aged 2–18 years old) were able to complete the test and have a measurable CS score. Good inter-examiner reliability when testing DH in adults and children was recorded, with the mean difference between examiners near zero.

As all questions in the survey had a section for additional comments, several participants noted that felt they were not aware of much literature on CS testing and felt more studies were needed on accuracy of current CS tests and normative values, these participants did not feel confident testing CS. There are some publications relating to CS testing and CS testing in children. A review paper from Milling et al. (2014) summarises the importance of CS testing in children and the literature present on different CS tests. Following this, there have been publications regarding normative values for PR (Mahjoob and Heydarian 2022) and normative values up to 36 months old for HH (Elgohary et al. 2017), providing predicted abnormal and normal values for CS tests which is important for clinical confidence of testing CS.

### Limitations of study

There are currently 1445 practising orthoptists in the United Kingdom, the response rate to the study was 5.8% which is low and limits any conclusions on orthoptic clinical practice regarding the use of CS tests. Not all answers were relevant to the questions asked and therefore, some responses to questions in the survey had to be eliminated. Some questions asked did not involve a free text box option preventing us from further exploratory analysis, particularly surrounding preferences for CS tests. Demographic data were not collected, therefore, may not be representative of a whole population.

One question in the survey asked participants if they feel there is a need for a new CS test for young children. This question has an element of bias as it assumes that tests for other populations are adequate e.g., adults with SEN, dementia patients etc.

## Conclusion

The result of the survey demonstrates the variability of CS testing currently in orthoptic practice in the UK. It also highlights the limited tests available currently for children for CS testing, which may be addressed by the addition of the new DH CS test. There is a need for increased knowledge of CS testing and current literature of CS tests to impact on clinical practice.
